# Changes in white matter microstructure and MRI-derived cerebral blood flow after 1-week of exercise training

**DOI:** 10.1038/s41598-021-01630-7

**Published:** 2021-11-11

**Authors:** J. J. Steventon, H. L. Chandler, C. Foster, H. Dingsdale, M. Germuska, T. Massey, G. Parker, R. G. Wise, K. Murphy

**Affiliations:** 1 Neuroscience and Mental Health Research Institute, School of Medicine, Cardiff University, Cardiff, UK; 2 Cardiff University Brain Research Imaging Centre, School of Psychology, Cardiff University, Maindy Road, Cardiff, CF24 4HQ UK; 3 School of Biosciences, Cardiff University, Cardiff, UK; 4 School of Physics and Astronomy, Cardiff University, Cardiff, UK

**Keywords:** Neurogenesis, Neurophysiology

## Abstract

Exercise is beneficial for brain health, inducing neuroplasticity and vascular plasticity in the hippocampus, which is possibly mediated by brain-derived neurotrophic factor (BDNF) levels. Here we investigated the *short-term* effects of exercise, to determine if a 1-week intervention is sufficient to induce brain changes. Fifteen healthy young males completed five supervised exercise training sessions over seven days. This was preceded and followed by a multi-modal magnetic resonance imaging (MRI) scan (diffusion-weighted MRI, perfusion-weighted MRI, dual-calibrated functional MRI) acquired 1 week apart, and blood sampling for BDNF. A diffusion tractography analysis showed, after exercise, a significant reduction relative to baseline in restricted fraction—an axon-specific metric—in the corpus callosum, uncinate fasciculus, and parahippocampal cingulum. A voxel-based approach found an increase in fractional anisotropy and reduction in radial diffusivity symmetrically, in voxels predominantly localised in the corpus callosum. A selective increase in hippocampal blood flow was found following exercise, with no change in vascular reactivity. BDNF levels were not altered. Thus, we demonstrate that 1 week of exercise is sufficient to induce microstructural and vascular brain changes on a group level, independent of BDNF, providing new insight into the temporal dynamics of plasticity, necessary to exploit the therapeutic potential of exercise.

## Introduction

Exercise stimulates neurogenesis, angiogenesis, and synaptogenesis, enhances neural plasticity, and ameliorates some of the deleterious morphological and behavioural sequelae of aging^1–3^. However, the mechanisms driving these changes are not fully understood, and there is a lack of clarity as to the relative sensitivity of physiological and biochemical parameters to the intensity, mode and duration of exercise. This knowledge could optimize therapeutic exercise prescription across different patient groups.

The hippocampus is a primary target for exercise effects in the brain, with exercise shown to alter hippocampal structure and/or function across both rapid and longer term timespans and age groups^4–8^. Brain-derived neurotrophic factor (BDNF) is a neurotrophin that supports neurogenesis, promotes neuronal survival and synaptic plasticity^9^, and has the highest expression levels in hippocampal neurons^10,11^. The expression of specific BDNF exons can be regulated by epigenetic mechanisms^12^, suggesting that environmental experiences can dynamically influence mature BDNF levels. In both young and aged rodents, exercise has been widely shown to increase BDNF expression in the hippocampus and cortical regions^13–16^, persisting for a number of weeks after exercise cessation^17^. In humans, both acute and chronic exercise modulates peripheral BDNF levels^18–20^, although the association between peripheral and cerebral BDNF concentrations is largely unknown, with some evidence from animal studies of a positive correlation^21,22^.

BDNF has been shown to induce myelination in white matter pathways in both animal and in vitro studies^23–25^. In humans, diffusion magnetic resonance imaging (MRI) can non-invasively assess brain white matter microstructure in vivo^26^. The most commonly reported microstructure metrics are derived from the diffusion tensor; fractional anisotropy (FA) indirectly reflects the extent to which the diffusion of water molecules in the brain tissue is preferentially hindered along one direction compared to others^27^ and mean diffusivity (MD) is the diffusivity average from the three eigenvalues of the tensor. Numerous studies in child and adult populations have examined the association between physical activity levels and/or aerobic fitness and diffusion tensor metrics. Results have been largely mixed^28^, with some reporting a positive association with FA in several white matter pathways^29^, including, but not limited to pathways associated with the hippocampus. Fewer studies have examined white matter changes following an exercise intervention^30–34^. A decrease in FA and an increase in MD has been observed in two separate studies following an aerobic exercise intervention (11 weeks and 6-months respectively), with changes observed across a number of distinct fibre pathways^30,32^. In contrast, an increase in FA in numerous white matter tracts has been reported following a combined aerobic and strength training intervention in both healthy participants and those with a diagnosis of schizophrenia^33^. Whilst an increase in FA and a decrease in MD are generally interpreted as an improvement in white matter microstructure in the literature, this inference should be treated with caution as tensor metrics lack biological specificity, with numerous factors affecting water displacement and contributing to the measurements, including fibre arrangement, degree of myelination, and axonal integrity. Advanced compartment models have been developed that isolate the signal contributions from different tissue compartments, and these have been shown to outperform the conventional tensor model^35^. To date, no study has examined the effect of an exercise intervention using compartment models of diffusion.

Exercise has also been shown to induce vascular plasticity, increasing the levels of angiogenic factors and inducing angiogenesis^36^, with converging research across species showing a hallmark effect of aerobic exercise on hippocampal perfusion^4,37^. Acute effects of exercise on cerebral blood flow (CBF) and cerebrovascular CO_2_ reactivity (CVR) appear to be dependent on the intensity, duration and mode of exercise and the measurement method^38–45^, yet the time dynamics involved in vascular plasticity are unclear. Using arterial spin labelling (ASL) MRI, a method which magnetically labels arterial blood water protons and measures CBF to a capillary bed in tissue, an increase in CBF up to 60 min following a single session of exercise was found specifically in the hippocampus, whilst no change was observed in CVR^6^, which is a measure of the change in CBF in response to a vasoactive stimulus such as carbon dioxide. In rodents, longer-term structural effects of exercise on the cerebral vasculature have been shown, with exercise increasing the total cortical capillary length, volume, and surface area^46,47^. A handful of human studies into the effects of longer-term (8–12 weeks) exercise training on the cerebrovasculature have produced mixed results, with both no change, an increase, and a decrease in regional CBF observed^4,48–51^; different sex and age distributions, patient populations, and measurement techniques likely contributed to the differences in observed effects. Non-MRI approaches have shown an elevation in CVR in the middle cerebral artery following a 12-week intervention^51^ whilst MRI-derived measures of CVR have not previously been acquired following an exercise intervention.

Thus, in summarising the temporal effects of exercise on the brain, the research literature shows acute exercise effects, captured during exercise itself and in the minutes and hours of exercise recovery, and long-term exercise effects, with 8–12 weeks the most common intervention duration. However, uncertainty remains regarding the magnitude and time course of potential cerebral adaptation due to methodological differences across studies. Moreover, the intermediate period between acute and chronic effects, namely the effect of days of exercise, has been largely neglected. An advantage of looking at short-term changes over days is that longer-term (weeks/months) exercise training also typically improves whole body energy metabolism, cardiovascular fitness and glucose homeostasis, and reduces adiposity and body mass^4,31,49^. This is problematic when aiming to isolate non-acute mechanisms and determine the direct effects on the brain. Understanding the time course of any changes will provide mechanistic insight and inform on the independence of cerebral changes.

Therefore, this study investigated the simultaneous changes in white matter microstructure and cerebral vasculature after 1 week of exercise. Our primary hypothesis was that 1 week of combined aerobic and resistance training would alter hippocampal blood flow and white matter microstructure in pathways associated with the hippocampus. We utilised a compartment model for diffusion-weighted MRI and hypothesized that the multi-compartment metric would be more sensitive than conventional diffusion tensor metrics in detecting exercise-induced white matter plasticity changes. Secondly, based on evidence that exercise modulates BDNF levels^18,52,53^, and BDNF induces both neurogenesis and angiogenesis^54^, we sought to investigate whether peripheral BDNF levels were associated with the effect of exercise on the brain.

## Results

Results on hardware variability and an assessment of change relative to literature-based reliability data are shown in the Supplementary Materials.

### Exercise intervention performance

100% of participants completed the 5 supervised exercise sessions. In total, participants cycled 60.1 ± 6.6 kms (daily average = 12.2 ± 1.6 km), with an average heart rate of 67.0 ± 5.8% maximum (127.7 ± 11.7 beats/min) and average revolutions per minute during cycling of 84.6 ± 19.0. IPAQ scores increased by 1592.3 ± 242.2 MET minutes in the week of the intervention (*p* = 1.24e−5).

### Exercise induced BDNF changes only in acute condition

Acutely, there was a significant increase in serum BDNF after completing the exhaustive fitness test (baseline = 20.8 ± 4.49 ng/ml, post-fitness test = 39.0 ± 4.49 ng/ml, t_23_ = 2.88, *p* = 0.008). Performance on the fitness test was not related to the change in serum BDNF (adj. R^2^ = 0.21, *p* = 0.12). After the 1-week exercise intervention, there was no difference in BDNF serum levels (17.6 ± 3.47 ng/ml) compared to baseline (19.6 ± 3.61 ng/ml, t_26_ = 0.41, *p* = 0.69). BDNF was missing from one participant due to issues with sample processing.

### Voxel-wise analysis: exercise induces changes in diffusion tensor metrics

Compared to baseline, an increase in FA and a decrease in RD were observed following the exercise intervention, in predominantly corresponding locations (Fig. 2*top panel,* *p* < 0.05). The differences were symmetrical across hemispheres and appeared to be localised to the corpus callosum, with some differences in the corticospinal tract, forceps minor, cingulum, anterior thalamic radiation, and uncinate fasciculus. There were no significant differences in the restricted signal fraction (FR) from the CHARMED framework (all voxels > 0.05).

### Tractography analysis: exercise-induced changes in restricted signal fraction

**Figure 1:**
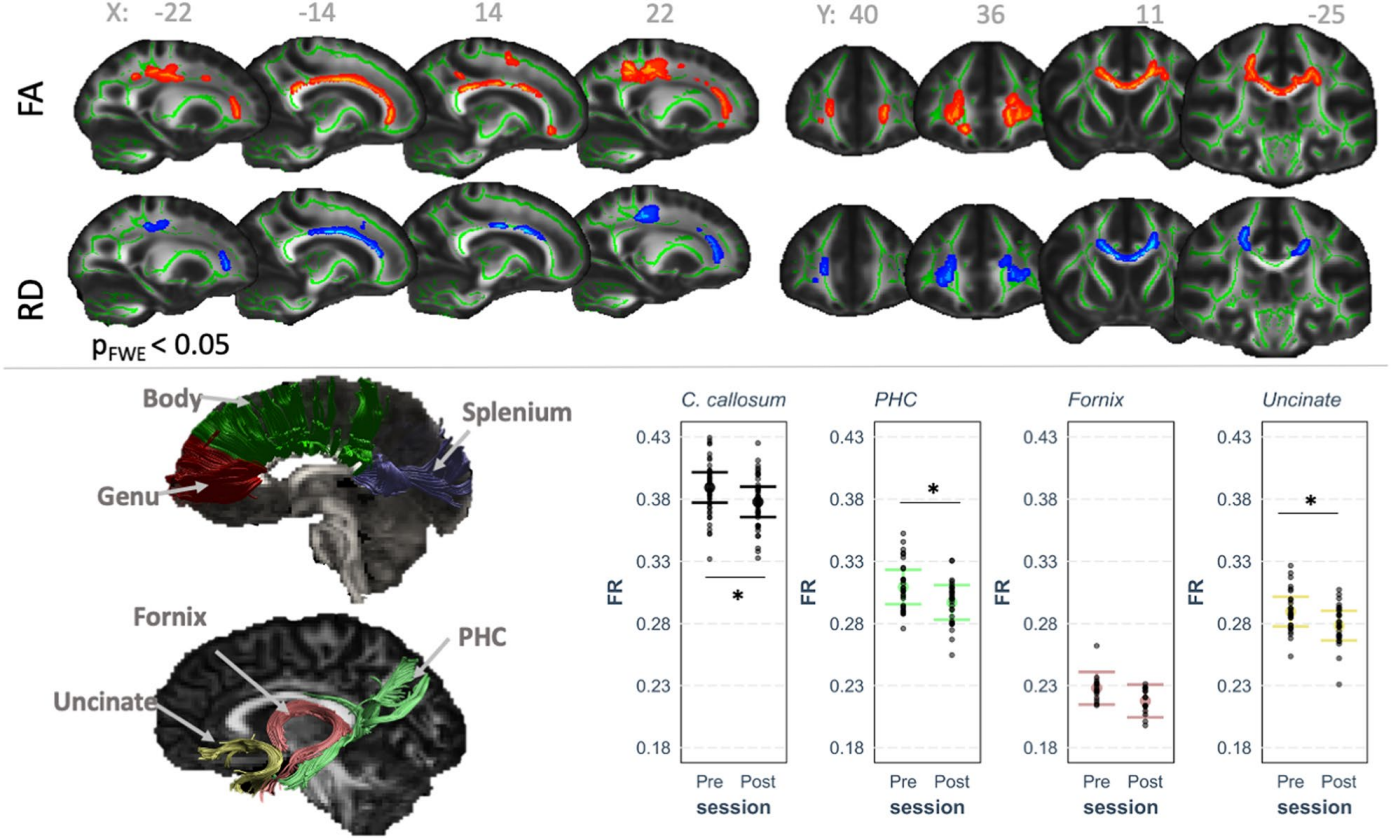
Diffusion MRI analysis showing exercise-induced differences. Top panel shows TBSS results for fractional anisotropy (FA) and radial diffusivity (RD) differences between the pre- and post-exercise intervention scans, clusters are displayed at *p* < 0.05, FWE-corrected (TFCE) and fattened with the “tbss_fill” script for the purpose of better visualization, shown against the the mean FA fibre skeleton (green) and overlaid on the FMRIB FA 1 mm template. Red represents a significant increase in values (post > pre) and blue represents a decrease (post < pre). Bottom panel shows representative tract segmentations for the corpus callosum (genu [red], body [green], splenium [purple]), uncinate fasciculus (yellow), fornix (pink), and parahippocampal cingulum (PHC; green), overlaid on the FA map. Graph shows the partial residual plot from the linear mixed effect model, showing the effect of exercise on restricted fraction (FR), with the effects of all the control variables accounted for, with error bars representing 95% confidence intervals and individual data points. The raw tractography FR data is graphically represented in Supplementary Fig. 5.

As shown in Table 1 and Fig. 1, exercise had a significant effect on FR in the corpus callosum (− 1.76%), parahippocampal cingulum (− 4.23%), and uncinate fasciculus (− 3.91%), with no changes observed for FA and MD in these tracts. There were no effects of exercise observed in the fornix for any of the diffusion parameters. Supplementary Figures 2 and 3 show the percentage change in FR in each fibre pathway for each participant, along with confidence intervals calculated using reference coefficients of variation from the published research literature. The relative change in BDNF levels across the 1-week intervention did not predict the change in FR in any of the tracts (all *p* > 0.05 uncorrected).

**Table 1:** Effect of exercise on diffusion MRI metrics.

Tract	Baseline		Post		Model statistics: fixed effect of exercise		
	Mean	SE	Mean	SE	β	t	*P* value
**FA**							
Corpus callosum	0.515	0.006	0.505	0.006	− 0.0093	1.59	0.921
PHC	0.348	0.006	0.341	0.006	− 0.0075	1.39	0.361
Fornix	0.367	0.006	0.361	0.006	− 0.0053	0.99	0.476
Uncinate	0.368	0.006	0.36	0.006	− 0.0088	1.58	0.361
**MD × 10**^**−3**^** mm**^**2**^**/s**							
Corpus callosum	0.738	0.006	0.743	0.006	0.0056	1.00	0.568
PHC	0.744	0.006	0.749	0.006	0.0050	0.80	0.568
Fornix	0.988	0.016	0.987	0.016	− 0.0004	0.04	0.973
Uncinate	0.725	0.005	0.73	0.005	0.0042	2.11	0.162
**FR**							
Corpus callosum	**0.409**	**0.005**	**0.398**	**0.005**	**− 0.0116**	**2.40**	**0.034**
PHC	**0.307**	**0.006**	**0.294**	**0.006**	**− 0.0124**	**2.31**	**0.034**
Fornix	0.228	0.006	0.218	0.006	− 0.0104	1.98	0.068
Uncinate	**0.281**	**0.005**	**0.270**	**0.005**	**− 0.0113**	**2.44**	**0.034**

Mean shown is the estimated marginal mean, adjusted for age and hemisphere.

Model statistics shown are from the linear mixed effect model for the fixed effect of exercise.

The *p* value is an FDR-adjusted *p* value.

Significant effects shown in bold.

*FA* fractional anisotropy, *MD* mean diffusivity, *FR* restricted volume fraction, *PHC* parahippocampal cingulum.

### Cerebrovascular measures: exercise increases hippocampal blood flow

All data and associated statistics are shown in Table 2. From the multi-TI ASL sequence, CBF in the hippocampus was 7.58% higher following the exercise intervention, with no change in CBF in the thalamus or globally in the grey matter (also see Supplementary Fig. 1 for absolute values for each region). Supplementary Fig. 4 shows the percentage change in hippocampal CBF for each participant, along with confidence intervals calculated using reference coefficients of variation from the published research literature. AAT was unchanged in the hippocampus, thalamus, and grey matter (*p* > 0.05). BDNF change following the intervention did not predict the change in CBF or AAT in the hippocampus (*p*_uncorr_ = 0.88 and 0.48, respectively), thalamus (*p*_uncorr_ = 0.67 and 0.84) or globally in the grey matter (*p*_uncorr_ = 0.71 and 0.37).

**Table 2:** Effect of exercise on cerebrovascular metrics.

	Baseline		Post		Model statistics: fixed effect of exercise		
	Mean	SE	Mean	SE	β	t	FDR-adjusted *p* value
Multi-TI ASL scan							
**CBF (ml/100 g/min)**							
Hippocampus	**47.50**	**1.91**	**51.10**	**1.91**	**3.63**	**2.39**	**0.043**
Thalamus	54.2	4.79	56.8	4.79	2.58	0.94	0.353
Grey matter	53.7	2.00	57.2	2.00	3.42	1.30	0.216
**AAT (seconds)**							
Hippocampus	1.15	0.014	1.16	0.014	0.006	0.44	0.897
Thalamus	1.29	0.012	1.29	0.012	− 0.002	− 0.13	0.897
Grey matter	1.33	0.007	1.34	0.007	0.003	0.42	0.678
Dual-calibrated fMRI scan							
**CVR (% CBF change/mmHg CO**_**2**_**)**							
Hippocampus	1.16	0.12	1.16	0.12	0.029	0.45	0.977
Thalamus	1.87	0.26	1.86	0.26	− 0.014	0.08	0.940
Grey matter	1.52	0.13	1.40	0.13	0.120	0.63	0.536

Mean shown is the estimated marginal mean, adjusted for age and hemisphere (and grey matter for regional cerebral blood flow [CBF]).

Model statistics shown are from the linear mixed effect model for the fixed effect of exercise.

The *p* value is an FDR-adjusted *p* value.

Significant effects shown in bold.

*AAT* arterial arrival time, *CVR* cerebrovascular reactivity.

From the dual-calibrated fMRI scan, baseline and post-exercise data was available for n = 10, due to technical and practical issues associated with conducting a respiratory challenge in the MRI environment. CVR in the hippocampus, thalamus, and globally in the grey matter were unchanged following 1 week of exercise (all *p* > 0.05). The relationship between CVR and BDNF change was not calculated due to the limited sample size.

## Discussion

This study provides a novel indication of the short-term effects of exercise on the neurotrophic factor BDNF, brain microstructure and cerebrovascular function, using a multi-modal MRI approach. Following a 1-week intervention combining aerobic and strength training, we found a group-level symmetrical change in diffusion tensor metrics in a number of white matter pathways, as well as tract-specific changes in the restricted fraction—a distinct axon-specific parameter—in the corpus callosum, parahippocampal cingulum and uncinate fasciculus. In line with our hypothesis, a selective group-level increase in hippocampal blood flow was observed following the intervention, whilst no change was observed in cerebrovascular reactivity (CVR) in the hippocampus.

For the first time, we used a multi-shell diffusion MR acquisition and compartment-specific modelling approach to examine exercise effects on white matter microstructure, going beyond conventional diffusion tensor indices to increase the biological specificity of the outcome measures. Using a TBSS voxel-based approach, 1-week of exercise training across five sessions was associated with changes in FA and RD symmetrically in voxels corresponding to the corpus callosum, anterior thalamic radiation, uncinate fasciculus, corticospinal tract, cingulum and forceps minor. The direction of the group-level changes are in agreement with two long-term intervention studies (> 3 months) which used a combined aerobic and strength training intervention and also reported an increase in FA and reduction in RD at follow-up localised across multiple fibre pathways^33,34^. Moreover, our results are partly in agreement with recent work by Lehmann et al.^55^, which examined the effect of seven cycling sessions at similar intensity to the current study, conducted over a 2 week period and also using TBSS. However, in contrast to our findings, Lehmann et al.^55^ found changes in MD and RD only in the right hemisphere, and not in regions corresponding to the corpus callosum. One notable problem with TBSS, and thus attempts to compare anatomically-specific findings across studies, is that the method is not tract specific as it cannot distinguish between different fibre orientations in voxels containing multiple fibre populations^56^.

To increase the anatomical specificity of our results, we additionally conducted a tractography analysis. Here, we observed a group-level selective reduction in the restricted fraction (FR), a metric obtained from the compartment-specific CHARMED framework^57^, in the corpus callosum, the uncinate fasciculus, and parahippocampal cingulum. No changes were found in the diffusion tensor indices, which supports work showing that CHARMED has increased sensitivity relative to a diffusion tensor model in characterising microstructural tissue changes arising during short-term neuroplasticity^58^. The reduction in FR indicative of exercise-induced structural remodelling is in the opposite direction to that hypothesised for a beneficial effect of exercise, suggestive of a short-term reduction in axon density. However, the biological processes underlying FR are not well-defined, affecting the interpretability; FR is normalised to the total water content, which means that changes in other tissue properties such as myelination, total water content, or micro-inflammatory processes and local complexity may also affect the degree of restrictance measured^59^.

When examining cerebral vascular effects, based on previous acute and long-term effects observed selectively in the hippocampus^4,6,37^, we took a region-of-interest approach and examined hippocampal CBF and global grey matter CBF, with the thalamus as a control region. Using an ASL sequence with multiple post labelling delays, to be able to examine AAT, we found a selective group-level increase in hippocampal CBF, with no change in AAT. A dual-calibrated fMRI scan was acquired to primarily measure cerebrovascular reactivity in the hippocampus, and globally in the grey matter. This scan exploits simultaneous measurements of BOLD and ASL signals during a hypercapnic-hyperoxic challenge and found no group-level changes in CVR in the hippocampus. This is in agreement with our previous findings acutely, where an increase in hippocampal CBF was observed immediately after exercise cessation, reflecting rapid redistribution of blood to this key area implicated in neuroplasticity, in the absence of a change in CVR^6^. We previously interpreted the lack of change in CVR as evidence against an exercise-induced mechanical vascular change. The observed increase in CBF is in agreement with previous work showing that a 2-week exercise intervention increased CBF, but in prefrontal brain areas using a whole-brain voxel-wise statistical analysis approach^55^. Maas et al.^4^ reported in a group of older adults participating in a 3-month exercise intervention that increasing fitness levels were positively associated with changes in hippocampal perfusion. Long-term exercise has been shown to increase capillary density in the hippocampus^36^ and hippocampal cerebral blood volume in humans^37^, yet many factors influence cerebral blood flow, hampering the ability to interpret a short-term increase in hippocampal CBF in the absence of a CVR change, and necessitating further work at this shorter time scale.

Acute exercise increases serum BDNF concentrations^60^, a finding that is replicated in this study, suggesting that our processing and analysis approach is sufficiently sensitive to detect exercise effects on BDNF. However, we found no changes in peripheral BDNF after the 1-week intervention, and no relationship between BDNF change and MRI measures. This is the first-time exercise effects on BDNF have been examined short-term in combination with MRI measures in humans and suggests 1-week is insufficient to induce short-term BDNF changes in adult males. This is in agreement with work in rodents, whereby a 3-day, 7-day, and 15-day exercise intervention was shown to induce protein changes associated with hippocampal synaptic and structural plasticity independent of BDNF upregulation^2^. Nevertheless, a caveat to this work is that peripherally measured BDNF may not accurately reflect central levels—whether BDNF crosses the blood–brain barrier is not currently established—with equivocal data on the correlation between concentrations of brain and peripheral BDNF^61–66^.

A methodological consideration in the interpretation of our results is that the results come from males only. Evidence from rodent studies suggests sex differences in the cerebral response to exercise, both for white matter microstructure and the cerebrovasculature^46,67^, thus sufficiently powered experiments examining sex differences in the human cerebral response to exercise are required. We recognise that the lack of a non-exercised control group is a caveat of our study design and attempted to mitigate this by (1) controlling for cyclical biological variation by testing participants at the same time of day and by having strict dietary and activity restrictions acutely prior to each scan, (2) using MRI sequences with good inter-session repeatability^68,69^, (3) using a scanner system with low levels of variability over the period of testing. A further caveat for the interpretation of our findings is that although we chose our exercise duration length to examine brain effects independent of other known physiological effects which occur over a longer time frame, including improved fitness, metabolism, and glucose homeostasis, and weight loss^70^, we did not measure any of these directly. Meanwhile, a strength of our study is the use of a linear mixed effect model which allowed us to model subject-related random effects (affecting pre- and post-exercise sessions equally) as well as the effects of a number of confounding variables. Nevertheless, our research findings concern the mean response to an exercise intervention, and do not extend to differences in the individual response to exercise training (subject-by-training interaction) nor to differences within the same participant on different training days^71^, which requires further research attention in the context of exercise-induced neuroplasticity.

To conclude, we have demonstrated that a 1-week intervention is sufficient to induce group-level microstructural and vascular changes commonly observed after much longer interventions (> 8 weeks) which provides insight into the temporal dynamics of plasticity. Changes were observed independent of BDNF and whilst controlling for acute effects by having participants refrain from exercise for 12 h prior to the follow-up scan. Distinguishing Distinction between acute, short- and long-term exercise effects is needed in order to realise the therapeutic potential of exercise for brain health.

## Method

**Figure 2:**
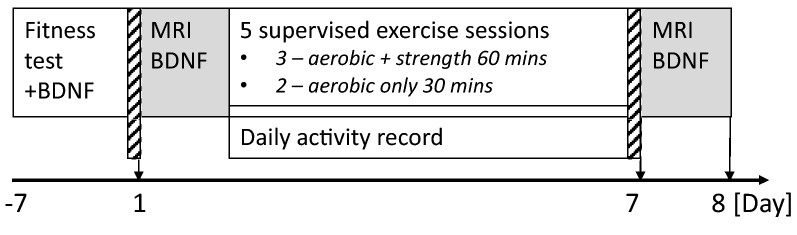
Study design. Brain-derived neurotrophic factor (BDNF) refers to the collection and sampling of blood serum for BDNF levels. For the fitness test, BDNF was sampled before and after the test. *MRI* magnetic resonance imaging.

The study design is detailed in Fig. 2. Analysis code and supporting data are available at https://doi.org/10.17035/d.2020.0117858659.

### Participants

15 healthy non-athlete males (29.7 ± 5.8 years old, range = 22–40 years), with no cardiac, vascular, respiratory or neurological pre-existing conditions, were recruited. We did not recruit females due to the short-term nature of the intervention and evidence of cerebrovascular changes at different phases in the menstrual cycle^72,73^. The inclusion and exclusion criteria are detailed in the Supplementary Information. Baseline demographics are shown in Table 3.

**Table 3:** Baseline demographics of male participants recruited.

	N = 15
Age (years)	29.7 ± 5.8
BMI (kg/m^2^)	26.1 ± 2.8
Heart rate (bpm)	65.1 ± 12.8
Systolic blood pressure (mmHg)	129 ± 8.6
Diastolic blood pressure (mmHg)	79.1 ± 6.6
IPAQ MET minutes	2673.5 ± 1900.5
IPAQ activity level classification^a^	1 inactive, 7 minimally active, 7 ‘HEPA’ active
Estimated VO_2_max ml/kg/min	39.8 ± 5.3

Data are expressed as mean ± SD.

*MET* metabolic equivalent, *HEPA* health enhancing physical activity: a high active category.

^a^Based on IPAQ scoring guidelines (www.ipaq.ki.se).

The study protocol was approved by the Cardiff University School of Psychology ethics committee (EC.17.03.14.4863) and was in accordance with the Declaration of Helsinki. All participants provided written informed consent.

### Exercise intervention

In line with the American College of Sports Medicine recommendations on the quantity and quality of exercise for healthy adults^74^, participants underwent a structured exercise program consisting of 5 supervised training sessions completed in a 1-week period combining aerobic and resistance exercise, in addition to maintaining their regular activities. During each session, participants performed moderate-intensity aerobic cycling. During three of the sessions, participants also performed resistance exercise. Each session was preceded by a 5-min warm-up and static stretching of the upper and lower limbs, neck, and trunk muscles, and was followed by a 5-min cool down. The training sessions were group sessions and supervised by a researcher. Heart rate (Polar S810, Polar, Finland), revolutions per minute, wattage, and distance were recorded at 5-min intervals during each session. The exercise program included the following: (1) *Warm-up and cool*-*down exercises*: Cycling on an upright ergometer at a low pace. (2) *Aerobic exercise (5 sessions)*: 25-min of moderate-intensity cycling on a Keiser M3 spin ergometer (Keiser M3 Indoor Cycle, Keiser, California, USA). Participants cycled at a speed and resistance level of their choice which maintained a heart rate at 50–75% of their age-predicted maximum. 3. *Resistance exercise (3 sessions, 2 sets per session)*: Body-weight exercises consisted of squats [12 repetitions], forward lunges [20 repetitions], push-ups [12 repetitions], gluteus bridges [12 repetitions], and tricep dips [20 repetitions].

### IPAQ

Self-reported physical activity data for the week preceding the intervention, and the week of the intervention, were collected using the IPAQ-S, which asks participants to report leisure time, work, domestic activities, and transport activities performed for at least 10 min during the last 7 days and across 3 intensities: walking, moderate, and vigorous. Using the instrument's scoring protocol^75^, total weekly physical activity was estimated by weighting time spent in each activity intensity with its estimated metabolic equivalent (MET) energy expenditure^76^.

### Baseline fitness

*V*O_2_max was estimated from a submaximal fitness test performed on a Lode cycle ergometer (Lode, Groningen, Netherlands). Prior to the test, participants were required to fast for 12 h overnight and abstain from alcohol and exhaustive exercise for 24 h, with all tests conducted between 0730 and 1100. Tests were terminated upon volitional exhaustion; additional details can be found in the Supplementary Information. Fitness tests were conducted in the 7-day period prior to the baseline MRI scan and a minimum of 24 h before the MRI scan. Fitness tests were not repeated following the intervention as *V*O_2_max was not expected to change in 1 week^77^.

### MRI acquisition

Participants were scanned at baseline before the commencement of exercise training, and after training, 1 week later. Scans were scheduled at the same time of day to remove the influence of diurnal effects, and participants were required to fast and abstain from caffeine for 6 h prior to the scan and refrain from exercise for 12 h. All MRI data were acquired using a Siemens MAGNETOM Prisma (Siemens Healthcare GmbH, Erlangen, Germany) 3 T clinical scanner with a 32 channel receive head coil. An estimate of the hardware variability from our scanner system was acquired on a phantom at weekly intervals during data collection and is shown in the Supplementary Materials.

For volumetric measurements and for image registration purposes, a 3D magnetization-prepared rapid gradient-echo (MPRAGE) sequence was acquired at both timepoints (1 mm slice thickness, 1.14 × 1.14 image resolution, TR/TE = 2100/3.2 ms). Multi-shell diffusion MRI data were collected using a spin-echo echo-planar high angular resolution diffusing imaging (HARDI) sequence with 6 b_0_ images, 30 diffusion directions at b = 1200 s/mm^2^ and 60 diffusion directions at b = 2400 s/mm^2^ sampled on a 128 × 128 matrix resulting in 2 mm^3^ voxel size (TE/TR: 67/9400 ms, *δ*/Δ: 17.9/32 ms, 80 slices, 80 mT/m gradient). The greater number of volumes at the higher diffusion weightings compensates for a lower signal-to-noise ratio and captures the higher angular resolution at higher *b*‐values^78^.

To measure CBF and arterial arrival time (AAT), a multiple post label delay (PLD) ASL sequence was acquired using a pCASL labelling scheme and echo-planar readout. The sequence implementation follows that of Okell et al.^79^ with pre-saturation, background suppression and a variable TR. However, the implementation used in this work did not include vessel selectivity. The labelling duration was 1.5 s, and data were acquired with 6 post labelling delays: 1.75 s, 2 s, 2.25 s, 2.5 s, 2.75 s, and 3 s. Partial Fourier of 6/8 and a TE of 13 ms were used to acquire 22 slices with an in-plane resolution of 3.3 × 3.3 mm and slice thickness 5 mm with a 1.25 mm slice gap. The first volume of our ASL sequence was a calibration image (M_0_) acquired for ASL quantification with pCASL labelling, pre-saturation, and background suppression switched off.

To quantify CVR, a dual excitation pCASL sequence^80^ was acquired. This scan included simultaneous acquisition of the blood-oxygen-level-dependent (BOLD) and ASL signals. Scan parameters included TR_1_: 3600 ms, TE_1_: 10 ms, TR_2_: 800 ms, TE_2_: 30 ms, GRAPPA acceleration factor: 3, slices: 15, slice thickness: 7 mm with a 1.75 mm slice gap, and in-plane resolution of 3.4 × 3.4 mm, with a tag duration of 1.5 s and post label delay of 1.5 s. The scan duration was 18-min and was acquired with interleaved periods of hypercapnia, hyperoxia, and medical air being delivered to the subjects according to the protocol previously detailed in Germuska et al.^81^.

End-tidal gases, P_ET_CO_2_ and P_ET_O_2_, were sampled from the volunteers’ tight-fitting facemask using a rapidly responding gas analyzer (AEI Technologies, Pittsburgh, PA, USA; PowerLab, ADInstruments, Sydney, Australia).

### Diffusion MR data analysis

Data were analysed using FMRIB Software Library (FSL, http://fsl.fmrib.ox.ac.uk) version 5.0.9^82^ and Explore DTI software^83^ (version 4.8.6). During preprocessing, the diffusion data were corrected for subject motion, eddy current induced geometric distortions, susceptibility-induced distortions and Gibbs ringing artefacts^84–88^. Using the b = 1200 s/mm^2^ shell only, a bi-tensor model was fit to separate diffusion properties of brain tissue from surrounding free water using the approach of Pasternak et al^89^. Next, the CHARMED model^57^ was fitted to the dual-shell HARDI data using in-house software written in Matlab R2015b. The CHARMED fitting procedure was based on non-linear least square estimation using a Levenberg–Marquardt optimization. The CHARMED model separates the contribution of the signal originating from the extra-axonal space and that originating from the intra-axonal space, by modelling the two distinct compartments with different analytical probability distribution functions^57^. From this, restricted non-Gaussian diffusion in the intra-axonal space was quantified with restricted signal fraction maps (FR, adapted from CHARMED to remove potential isotropic partial volume contamination) using the fibre orientation density function (fODF) peaks.

#### TBSS

For the voxel-wise statistical analysis of diffusion MR data, FA maps were inputted into the Tract-Based Spatial Statistics (TBSS) package of FSL^90^ and the TBSS pipeline was applied using the recommended parameters. Based on evidence showing improved alignment^91^, we constructed a study-specific template for the registration of FA images across subjects. DTI measures, along with the CHARMED metric FR, were projected onto a mean FA tract skeleton which represented the centre of all tracts common to the group and was thinned using an FA threshold > 0.2. A general linear model was used within a voxel-wise, permutation-based, non-parametric statistical framework to test for differences before and after the exercise intervention controlling for multiple comparisons across clusters using Threshold-Free Cluster Enhancement (TFCE). We employed 10,000 permutations, and a corrected voxel-wise *p* value < 0.05 was considered statistically significant. Automatic localization of significant clusters was performed using the Jülich Histological Atlas^92^. As a data quality assurance measure to confirm the significant skeleton voxels were derived from the correct tract-centre point in all participants, skeleton voxels were inverse warped into each subject's native space through an inverse nonlinear registration and visually inspected.

#### Tractography analysis

In regions where pathways of different structures merge, it becomes difficult to resolve the anatomical specificity of findings with TBSS^56^. Thus, we reconstructed fibre tracts across the whole brain, applying multi-shell constrained spherical deconvolution^93^ to obtain voxel-wise estimates of the fODF with maximal spherical harmonics order lmax = 6. Seed points were evenly distributed across vertices of a 2 mm isotropic grid and propagated in 1 mm steps with a streamline length constraint of 30–300 mm. Tracking was terminated if streamlines exceeded a 45° maximum curvature angle in successive steps or if the fODF fell below 0.05.

Based on hypotheses specific to the hippocampus^29,94, 95^, we segmented the corpus callosum (genu, body and splenium separately, see Fig. 1) along with the three major tracts associated with the hippocampus: the parahippocampal cingulum, the fornix, and the uncinate fasciculus (Fig. 1). To segment the corpus callosum, fornix and the uncinate fasciculus, the approach of Parker et al.^96^ was used, applying a shape model for each tract based on principal component analysis. The shape and location models were trained on manually reconstructed tracts from 20 randomly selected datasets to automatically extract the tracts of interest at baseline and post-exercise from all patients and controls. All automatically segmented tracts were visually inspected in three dimensions and spurious streamlines inconsistent with tract anatomy were removed if necessary. The shape models can be accessed here: https://doi.org/10.17035/d.2020.0117858659. The parahippocampal cingulum was manually segmented using the approach of Jones et al.^97^, due to poor performance of the automated approach in this dataset.

### CBF quantification

Quantification of CBF and AAT—the time taken for blood to travel from the labelling plane to the tissue^98,99^—were performed using in-house analysis pipelines, AFNI version 16.2.18 (http://afni.nimh.nih.gov)^100^ and FSL version 5.0^82^. To correct for motion, time-series images were spatially registered using the 3dAllineate function in AFNI. Images were registered to the mean volume of the condition and an affine registration was performed with 6 degrees of freedom, a one pass alignment strategy and local Pearson correlation cost function. Non-brain tissue was removed from images using the brain extraction tool in FSL^101^. CBF quantification was performed using the BASIL toolkit in FSL^82,102^ on a voxel-by-voxel basis using a two-compartment model. The M_0_ image was spatially smoothed using a 5 mm Gaussian kernel, and the relative CBF image was then normalized by this smoothed M_0_ image using the labelling efficiency values as recommended by Alsop et al.^103^.

### Vascular reactivity quantification

Using AFNI^100^, image time series were motion corrected using 3dvolreg and brain extracted using 3dAutomask. Interpolated surround subtraction was performed on the ASL tag-control image time series to yield a perfusion-weighted time series. To quantify CBF, the signal from CSF was measured from the fully relaxed scan to estimate the M_0_ of blood and CBF values were calculated using a single compartment model^104^ and using the labelling efficiency values and blood T_1_ values quoted by Alsop et al.^103^. The end-tidal carbon dioxide (PETCO_2_) trace was convolved with a single hemodynamic response function and fitted to the perfusion image time series in each subject, using AFNI’s 3dDeconvolve to obtain a whole brain CVR map, measured as % CBF signal change per mmHg PETCO_2_ change. A grey matter mask was created from the session-specific T_1_-weighted image (see below).

### Region-of-interest analysis: CBF, AAT, CVR

Using the whole-brain CBF, AAT and CVR maps generated, regional changes were assessed in the hippocampus, the a priori hypothesised area of exercise-induced effects^5,6,8,37^. The thalamus was selected as a control region, having previously been shown to be unaffected by exercise^5,6^. ROIs were derived from the Harvard–Oxford subcortical structural atlas to which the participants’ CBF, AAT and CVR maps were registered (see Supplementary Information for additional details). To account for partial volume effects, a probabilistic partial volume tissue segmentation (FSL’s FAST) was conducted on the average T_1_-weighted image and the perfusion data were masked for partial volume grey matter values greater than 50%. To estimate blood flow per unit grey matter, the CBF data were divided by the partial volume image. Median CBF, AAT and CVR were extracted from each ROI, and globally in the grey matter, for statistical analysis.

### BDNF blood sampling and biochemical analysis

For acute exercise effects, a venous catheter was placed in the right forearm prior to the fitness test. Fifteen minutes after placement of the catheter, a baseline blood sample was collected. Post-exercise blood samples were collected after the fitness test and cool-down period. For effects of the 1-week intervention, a venous blood sample was taken immediately prior to the MRI scan at baseline and post-intervention.

Serum was generated by allowing samples to incubate at room temperature for 1 h, followed by at least 1 h at 4 °C. Samples were then centrifuged at 2000 rpm for 10 min before separation of serum and storage at − 80 °C until analysis. Although samples collected before and after acute exercise had variable incubation lengths during the 4 °C incubation, pre- and post-exercise samples from the same participant were time-matched.

Serum BDNF levels were measured as described previously^105^. BDNF antibodies #1 and #9^106^ were conjugated with biotin (sulfo-NHS-LC-Biotin, ThermoFisher Scientific) and horseradish peroxidase (HRP, peroxidase labelling kit, Roche) following manufacturers’ instructions.

NeutrAvidin-coated plates (ThermoFisher Scientific) were washed twice with Buffer A (0.1% Triton X-100 in 0.1 M phosphate buffer: 0.1 M KH_2_PO_4_, 0.1 M Na_2_HPO_4_, pH 7.6; Sigma) before coating with 13 μg/ml biotin-conjugated BDNF mAb-#1 for 2 h. Plates were then washed three times with Buffer B (Buffer A with 1% bovine serum albumin; Sigma), and wells refilled with 150 μl Buffer A. 50 μl of either recombinant BDNF standard or serum sample, diluted in Buffer B, was then added to wells and the plate incubated for 3 h on a rotary shaker. After another three washes with Buffer A, wells were incubated with BDNF mAb-#9 at a 1:800 dilution for 3 h on a rotary shaker. Following three final washes with Buffer A, a chemiluminescent substrate (Chemiluminescence ELISA substrate, Roche) was added to wells and the plate immediately analysed using a microplate reader (FLUOstar OMEGA, BMG Labtech).

### Statistical analysis

An initial quality assessment examined any statistical outliers, defined as more than 3 standard deviations from the group mean, and removed them if found to be spurious (e.g. biologically implausible). To avoid biasing the results, all participants were included in the statistical analysis, including when missing data was present.

For the TBSS analysis of the diffusion MR data, voxel-wise analyses of the fibre skeleton^107^ comparing pre- and post-exercise sessions was performed using non-parametric permutation testing with n = 5000, corrected for multiple comparisons and TFCE^108^ with *p* = 0.05 as the threshold for significance, using the randomise tool in FSL^109^.

For the tract-based diffusion analyses, CBF, AAT and CVR analyses, a linear mixed effect model was fitted using LMER^107^, from the statistical software package R (version 1.1.463, GNU General Public License). To address heterogeneity bias and avoid violating an assumption of mixed models^110^, age was demeaned and added to the model along with hemisphere. To reduce the number of multiple comparisons in the diffusion MR analysis, FA, MD and FR were assessed. Where a significant effect was observed in FA or MD, the component eigenvalues, axial diffusivity, and radial diffusivity (RD), were then assessed.

To assess the relationship between BDNF and MRI measures, the relative change between post-intervention and the baseline session was calculated for each measure and a linear model fit, with age included.

For all analyses except TBSS, a false discovery rate (FDR) of q = 0.05 was used to correct for multiple comparisons, using the Benjamini–Hochberg procedure^111^.

## Supplementary Information

Supplementary Information.
