# Supplementary material for: Changes in white matter microstructure and MRI-derived cerebral blood flow after 1-week of exercise training
Source: Sci Rep. 2021 Nov 11;11:22061. doi: 10.1038/s41598-021-01630-7 (PMC8586229; doi:10.1038/s41598-021-01630-7)
Supplement: Supplementary file 1 — Supplementary Information. [file 41598_2021_1630_MOESM1_ESM.pdf]

Changes in white matter microstructure and MRI-derived cerebral blood flow after one-week of exercise training

Steventon, J. J.* [1, 2], Chandler, H. L.†[2], Foster, C. †[2], Dingsdale, H. †[3], Germuska, M. [2], Massey, T.[1], Parker, G.[2] , Wise, R. G.[2], Murphy, K. [2,4]

**Corresponding author*. Email: steventonjj@cardiff.ac.uk. Contact address: Cardiff University Brain Research Imaging Centre, Cardiff University, Maindy Road, Cardiff. CF24 4HQ

†*contributed equally to the work*

1. *Neuroscience and Mental Health Research Institute, School of Medicine, Cardiff University, UK*
2. *CUBRIC, School of Psychology, Cardiff University, UK*
3. *School of Biosciences, Cardiff University, UK*
4. *School of Physics and Astronomy, Cardiff University, UK*

Supplementary Information

Participants

Participants were recruited based on the following inclusion and exclusion criteria.

Inclusion criteria:

- Male (scientific justification: to reduce variance, based on gender differences in exercise physiology)
- Aged 18-45 years old (scientific justification: males aged over 45 years old have an increased cardiovascular risk factor according to the American College of Sports Medicine guidelines)
- Able to understand and communicate in spoken English *(for consent purposes)*
- If regular medication is taken, the medication regime must have been stable for four weeks prior to initiation of the study

Exclusion criteria:

- Any physical or psychiatric condition that would prohibit the participant from completing the intervention or the full battery of assessments including previous spinal surgery or infection, or inflammatory disease of the spine, or arthritis
- A (self-reported) blood-borne disease and/or haemophilia
- history of claustrophobia (due to MRI)
- Inability to independently use the exercise bike
- Currently actively involved in any interventional trial or within four weeks of completing an interventional trial
- MRI contraindications (e.g., a pacemaker)
- Any known neurological condition / abnormality
- have now or have had in the past cardiac (heart), vascular (blood vessel) or respiratory/pulmonary (breathing/lung) conditions, including high blood pressure,
- experience dizziness or fainting on a regular basis
- you suffer from either asthma or diabetes mellitus (due to respiratory gas manipulation and/or exercise component)
- smoker (due to respiratory gas manipulation)

- have taken any illicit drugs in the last 4 weeks
- History of adverse response/s (including fainting) following exercise
- Having donated blood in the 4-week period prior to the first research visit, or planning to donate blood in the 4 weeks following the final research visit

Baseline Fitness

The fitness test began with a 1-minute warm up at 25 watts. The test began at 50 watts and was increased by 15 watts every 60 seconds while participants maintained a target constant RPM of 70. The test was terminated if RPM dipped below 60 for >10 seconds. Borg ratings of perceived exertion (RPE) for legs and breathing were recorded every minute using the CR10 scale (Borg & Kaijser, 2006). Capillary blood lactate and haemoglobin levels (Hb) were measured from the earlobe at rest and at the point of test termination (100% exertion). Blood pressure (BP) and heart rate (HR) were recorded at baseline, at the end of each test step and at test termination using the MEDRAD system (MEDRAD, Pittsburgh, PA) and Polar FT1 heart rate monitors (Polar, UK) respectively. Participants were encouraged to cycle until heart rate exceeded 95% of the age predicted maximum.

Region-of-interest analysis: CBF, AAT, CVR

Registration was conducted using FSL FLIRT with 12 DOF and trilinear interpolation. The registration involved transforming the participants averaged (between the two sessions) T1 MPRAGE to MNI space using a standard template. Functional data were then transformed to T1 space and output matrices were concatenated and used to take the data to MNI space. From here the inverse matrix was used to transform the ROIs back to subject space.

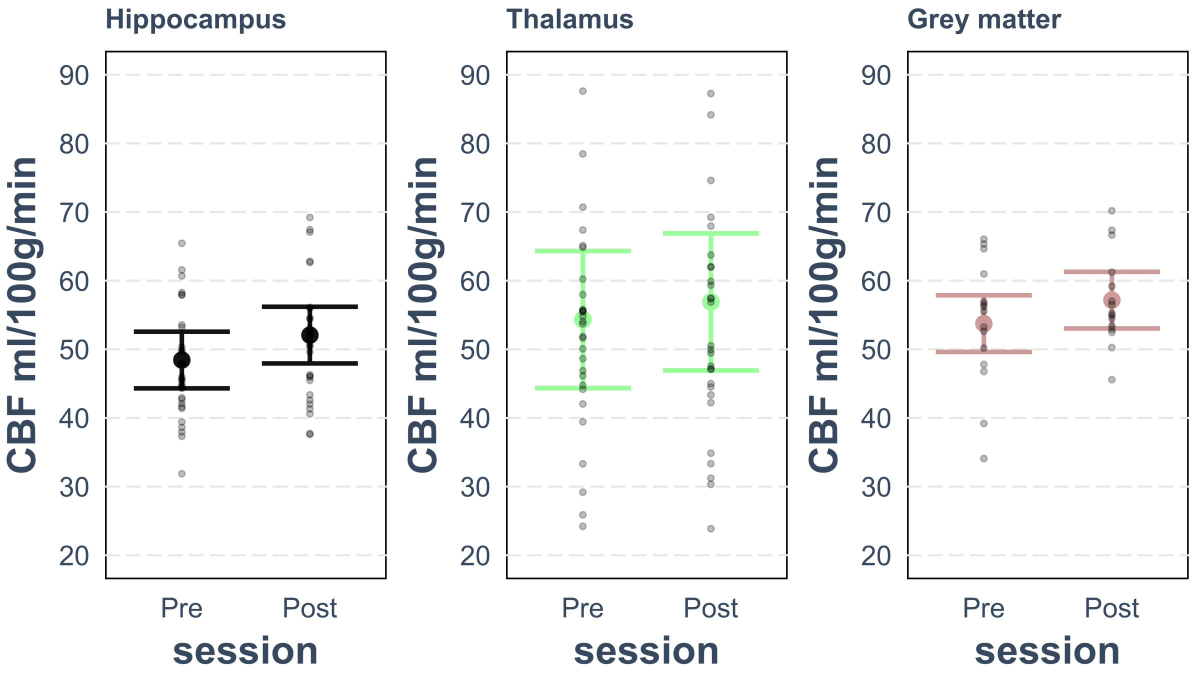

Supplementary Figure 1. Graph shows the effect of exercise on cerebral blood flow (CBF)) from the linear mixed effect model, with error bars showing 95% confidence intervals, with individual data points shown.

Stability of scanner system over intervention period

The mean SNR (mean signal in a region of interest in the centre of the image / standard deviation of region of noise) of the Prisma scanner used during the scan period was 552.8 and the coefficient of variance of the SNR over the same period was in the order of 3%, which represents a high stability well within 2*std.

The mean signal-to-fluctuation noise (SFNR; defined as the mean signal intensity of EPI timeseries divided by the standard deviation of the total noise within the of EPI timeseries) was 1380.4 with a coefficient of variance of 2.6%, thus there is no evidence of a systematic change in the scanner stability over the time course of the study.

Assessment of change relative to literature-based reliability data

To assess whether the observed significant changes observed in the various MR metrics exceeded the within-subject standard deviation expected, the typical error of the various metrics were calculated using coefficient of variation data from the research literature and the approach recommended by Swinton et al.[1], as shown in Supplementary Table 1. As can be seen in Supplementary Figures 2-4, the change scores +/- the adjusted true score change confidence intervals lie outside the zero-line in the vast majority of subjects, indicating that the observed results are consistent and robust.

High reproducibility of several microstructural MRI measures across scan sessions has been shown by intra-class correlation coefficients and coefficients of variation from multi-shell and multi-direction encoded diffusion datasets repeated 5 times over a 2-week period[2]. High reproducibility was reported for both tensor and CHARMED based metrics acquired in white matter tracts using a tractography-based analysis approach (coefficient of variation =0.2-2.1% depending on metric and tract) and at a voxel level; FA (shown to be elevated in our study) was found to be the most reproducible metric ($r >$ 0.95) at the voxel level, and RD (significantly reduced in our study) similarly showed good performance ($r > 0.84$).

Supplementary Table 1. Values used for calculating true score confidence intervals (according to Swinton et al. 2018). FR: restricted fraction. CBF: cerebral blood flow. CV: coefficient of variation.

Region	Metric	Literature CV (%)	Observed Score	Typical Error	Reference
Corpus callosum (averaged)	FR	1.67	-2.623	-0.044	Koller et al.[2]
Genu	FR	1.67	-1.764	-0.029	
Body	FR	1.67	-3.708	-0.062	
Splenium	FR	1.67	-2.396	-0.040	
Uncinate	FR	1.67	-3.655	-0.061	Koller et al.[2]
Parahippocampal Cingulum	FR	1.67	-1.803	-0.030	Koller et al.[2]
Fornix	FR	1.67	-4.214	-0.070	Koller et al.[2]
Hippocampus	CBF	5.33	9.055	0.483	Mezue et al.[3]

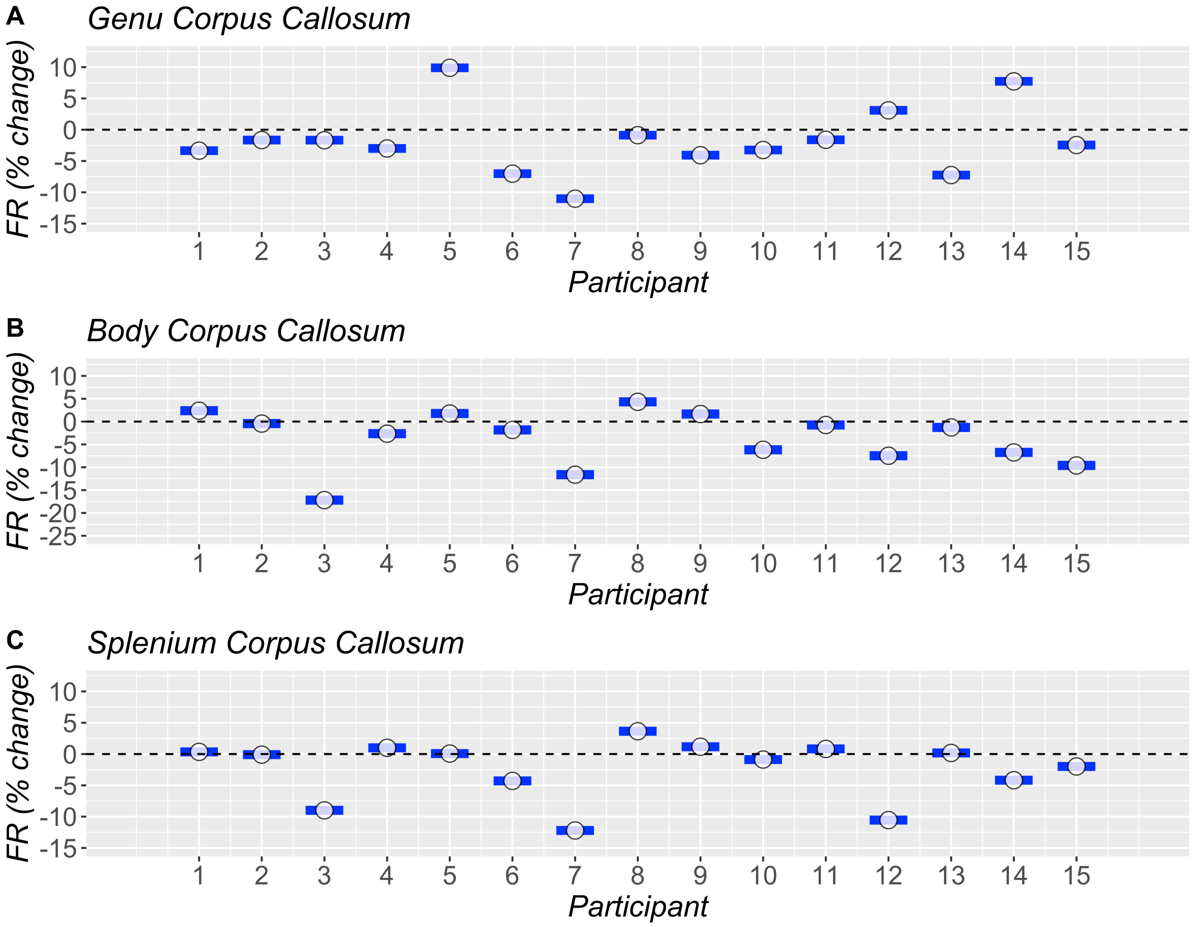

Supplementary Figure 2 Change in tissue microstructure in the corpus callosum observed using tractography segmentation. Data shown are percentage change in restricted volume fraction (FR) following the exercise intervention, shown for each subject and callosal segment (A-C). Error bars (shown in blue) are adjusted true score change confidence intervals (95%), calculated according to Swinton et al. (2018) using reference coefficients of variation values from Koller et al. (2021). The horizontal black dashed line represents the zero-line indicating no post/pre change.

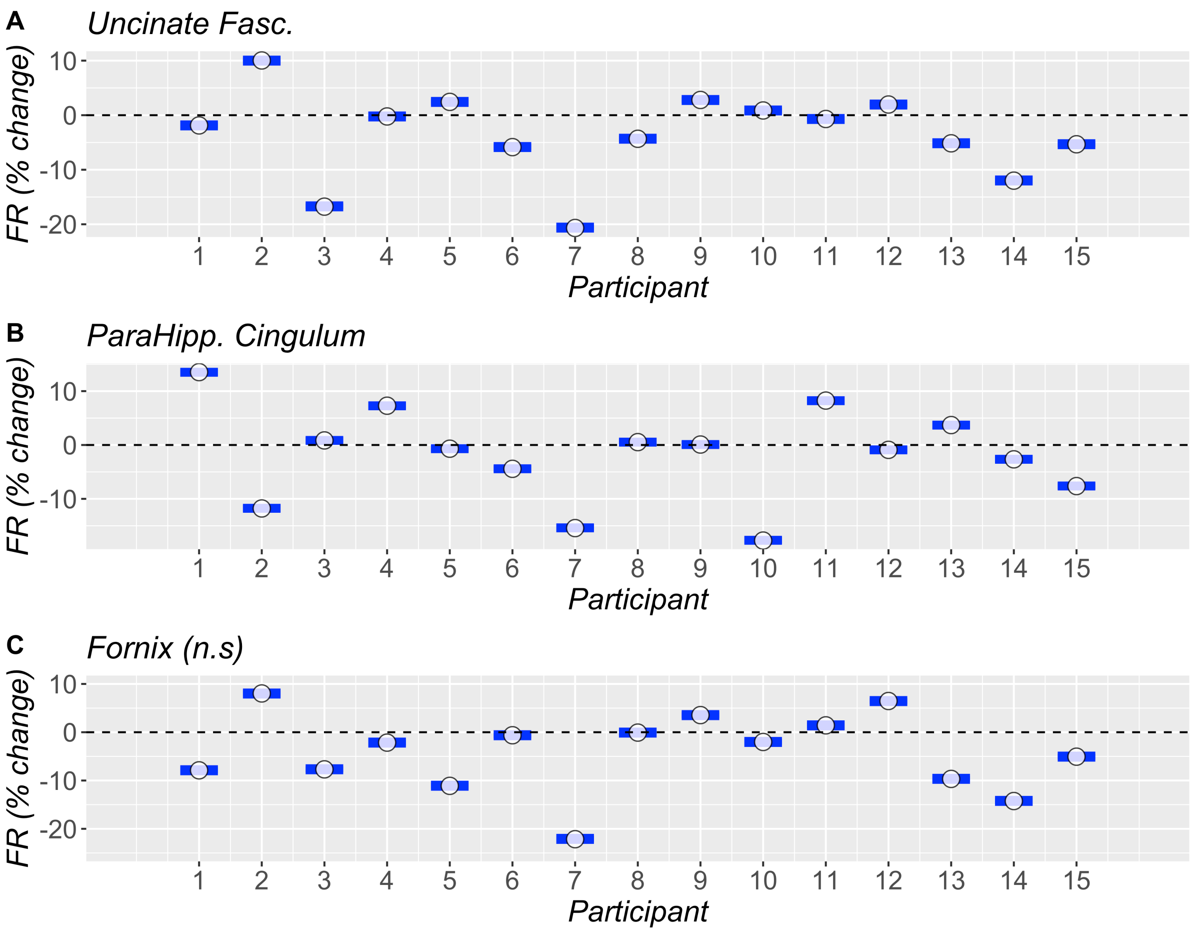

Supplementary Figure 3 Change in tissue microstructure in limbic white matter tracts observed using tractography segmentation. Data shown are percentage change in restricted volume fraction (FR) following the exercise intervention, shown for each subject in the uncinate fasciculus (A), parahippocampal cingulum (B), and fornix (C). Error bars (shown in blue) are adjusted true score change confidence intervals (95%), calculated according to Swinton et al. (2018) using reference coefficients of variation values from Koller et al. (2021). The horizontal black dashed line represents the zero-line indicating no post/pre change.

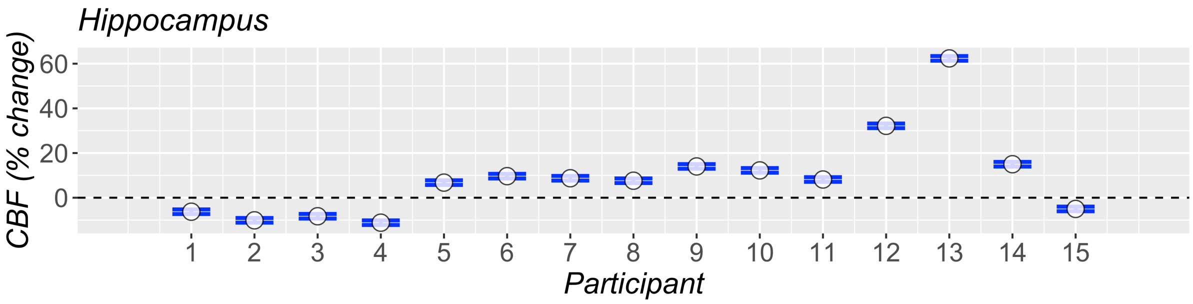

Supplementary Figure 4 Change in hippocampal blood flow. Data shown are percentage change in CBF following the exercise intervention. Error bars (shown in blue) are adjusted true score change confidence intervals (95%), calculated according to Swinton et al. (2018) using reference coefficients of variation values from Mezue et al. 2014. The horizontal black dashed line represents the zero-line indicating no post/pre change.

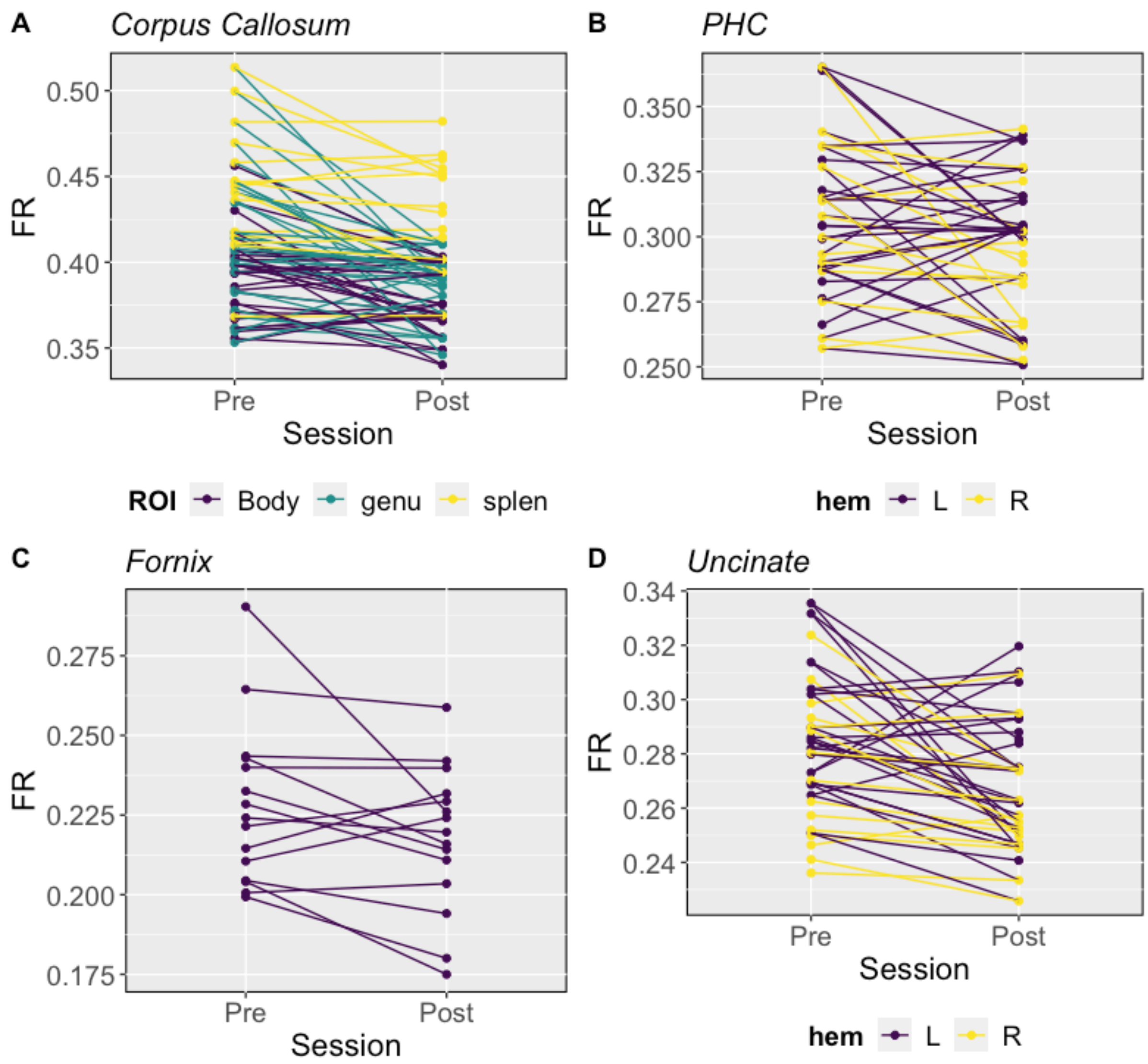

Supplementary Figure 5. Raw restricted volume fraction (RF) data for each tract pathways examined, shown for each participant. In [A], data from the three callosal segments (body, genu, splenium) is shown. Hem: hemisphere; L: left; R: right. PHC: parahippocampal cingulum.

Supplementary references

1. Swinton, P. A., Hemingway, B. S., Saunders, B., Gualano, B. & Dolan, E. A Statistical Framework to Interpret Individual Response to Intervention: Paving the Way for Personalized Nutrition and Exercise Prescription. *Front. Nutr.* **5**, 41 (2018).

2. Koller, K. *et al.* MICRA: Microstructural image compilation with repeated acquisitions. *Neuroimage* **225**, 117406 (2021).

3. Mezue, M. *et al.* Optimization and reliability of multiple postlabeling delay pseudo-continuous arterial spin labeling during rest and stimulus-induced functional task activation. *J. Cereb. Blood Flow Metab.* **34**, 1919 (2014).
